# Variation in stroke care at the hospital level: A cross-sectional multicenter study

**DOI:** 10.3389/fneur.2022.1004901

**Published:** 2022-10-13

**Authors:** Charlotte Lens, Ellen Coeckelberghs, Deborah Seys, Jelle Demeestere, Caroline Weltens, Kris Vanhaecht, Robin Lemmens

**Affiliations:** ^1^Department of Public Health, Leuven Institute for Healthcare Policy, KU Leuven—University of Leuven, Leuven, Belgium; ^2^Department of Neurology, University Hospitals Leuven, Leuven, Belgium; ^3^Department of Neurosciences, Experimental Neurology, KU Leuven—University of Leuven, Leuven, Belgium; ^4^VIB, Laboratory of Neurobiology, Center for Brain & Disease Research, Leuven, Belgium; ^5^Department of Oncology, University Hospitals Leuven, Leuven, Belgium; ^6^Department of Quality, University Hospitals Leuven, Leuven, Belgium

**Keywords:** stroke, variation, care process, adherence, guidelines

## Abstract

**Introduction:**

Stroke is one of the leading causes of mortality and disability. Improving patient outcomes can be achieved by improving stroke care and adherence to guidelines. Since wide variation in adherence rates for stroke guidelines still exists, we aimed to describe and compare stroke care variability within Belgian hospitals.

**Materials and methods:**

An observational, multicenter study was performed in 29 Belgian hospitals. We retrospectively collected patient characteristics, quality indicators, and time metrics from the last 30 consecutive patients per hospital, diagnosed with ischemic stroke in 2019 with structured questionnaires. Mean adherence ratios (%) ± SD (minimum – maximum) were calculated.

**Results:**

We analyzed 870 patient records from 29 hospitals. Results showed large inter- and intrahospitals variations in adherence for various indicators. Almost all the patients received brain imaging (99.7%) followed by admission at a stroke unit in 82.9% of patients. Of patients not receiving thrombolysis, 92.5% of patients were started on antithrombotic drugs. Indicators with moderate median adherence but large interhospital variability were glycemia monitoring [82.3 ± 16.7% (26.7–100.0%)], performing clinical neurological examination and documentation of stroke severity [63.1 ± 36.8% (0–100%)], and screening for activities of daily living [51.1 ± 40.3% (0.0–100.0%)]. Other indicators lacked adequate adherence: swallowing function screening [37.0 ± 30.4% (0.0–93.3%)], depression screening [20.2 ± 35.8% (0.0–100%)], and timely body temperature measurement [15.1 ± 17.0% (0.0–60%)].

**Conclusion:**

We identified high adherence to guidelines for some indicators, but lower rates with large interhospital variability for other recommendations also based on robust evidence. Improvement strategies should be implemented to improve the latter.

## Introduction

Physicians and other healthcare professionals aim to improve the quality of care by implementing and adhering to evidence-based guidelines. However, evidence shows that only 55% of patients receive recommended care, resulting in a gap between guidelines and daily clinical practice (1–4). This may interfere with patients receiving optimal medical treatment, as described in clinical guidelines, and may affect outcomes (5).

Despite positive associations between implementing evidence-based care and outcomes of patients after ischemic stroke (IS), unwarranted variation still exists (6–10). Organized stroke care reduces mortality and disability in patients with IS, by implementing effective strategies in stroke management and treatment (10–12). The beneficial effect of organized multidisciplinary stroke care on patient outcomes was shown in the Quality in Acute Stroke Care study (13). In this study, a protocol for assessment and management of body temperature, hyperglycemia, and dysphagia (FeSS protocol: Fever, Sugar, and Swallowing) in the first 72 h after admission was developed. Compliance with this protocol resulted in a significant reduction of 90-day mortality and dependency in patients with IS (13–15).

Care processes and outcomes are frequently evaluated based on data from regional or national stroke registers (6, 11, 16–18). The obtained results give insights in overall quality of care, which is valuable to assess merits of issued policies within a region or country. However, differences in the care process between hospitals remain mostly unknown. Translation of evidence-based guidelines into clinical practice starts with providing frameworks, but the success of the implementation depends on hospital environment (5, 19–22). Differences in this environment may impact adherence to recommendations and, thereby, variation in outcome (23). There is limited data on comparing care processes on the hospital level.

The aim of this multicenter study was to investigate the variation in care for patients with IS. We prespecified process indicators (PIs) and quality indicators (QIs) in the stroke care pathway from admission at the emergency department (ED) to hospital discharge, based on current evidence (24, 25).

## Materials and methods

### Design, setting, and population

An observational, cross-sectional, and retrospective multicenter study in 29 Belgian hospitals was performed. Hospitals were recruited through the Belgian-Dutch Care Pathway Network and/or Flemish Hospital Network KU Leuven. All the Flemish hospitals members of one of these two organizations were invited to participate in this study. A total of 37 hospitals agreed to participate and were invited for participation in this study. To ensure anonymity of the hospitals, a randomization number was assigned to each hospital throughout this article. Within the participating hospitals, 30 patients consecutively discharged (or who died in hospital) before 31 December 2019 were included, if fulfilling following criteria: (1) age ≥ 18 years and (2) admitted to the ED and diagnosed with IS. In case of death, the date of death was recorded as the date of discharge. Patients were excluded if treated with mechanical thrombectomy, since most of these patients would not undergo the full pathway at one hospital, as a result of transfer to a comprehensive stroke center. Data were collected from patient records by a local study coordinator in each hospital using standardized questionnaires. Data not retrievable or available in the patient record were reported as “no information in record.”

### Variables

Table 1 summarizes the included QI, the recommendations which they are based on and their level of evidence. For the development of this set of QI, guidelines describing the care process for patients with ischemic stroke were collected and screened (24–26). From these guidelines, we selected the recommendations applicable to the care process from arrival to the ED until discharge from neurology department. This selection was shared with the participating hospitals during an online meeting and circulated by email. Neurologists, members of the multidisciplinary stroke team, and healthcare quality managers were given the opportunity to give their feedback on this selection and to make any necessary adjustments. Based on this feedback, a definitive list of recommendations was defined. This resulted in a set of 12 QI (Table 1) and 6 PI. The Joint Commission on Accreditation of Healthcare Organizations (JCAHO) defines QI as “quantitative measures that can be used to monitor and evaluate the quality of important governance, management, clinical, and support functions that affect patient outcomes” (27, 28). “Adherence to QI” refers to adherence to the evidence-based guidelines as quantified *via* quality indicators.

**Table 1:** Selected QI with corresponding recommendation from guidelines and level of evidence (LOE).

**QI**	**Recommendation from guidelines**	**LOE**
ADL-screening	A formal assessment of the activities of daily living should be performed in every patient with IS.	B-NR^24^
No administration of preventive antibiotics	Prophylactic antibiotics should not be used routinely in patients with IS.	A^24^
Admission to specialized SU	Patients need to be admitted to a specialized SU.	A^24^
Antithrombotic administration	Antithrombotic should be administrated as primary treatment if thrombolysis was contraindicated.	A^24^
Body temperature measurement	Measurement of body temperature every 4 h during the first 72 h after admission.	B^39^/FeSS^13^
Brain imaging	Brain imaging should be performed in all patients with suspected stroke.	A^24^
Cardiac monitoring	Cardiac monitoring should be performed during hospitalization.	B-NR^24^
Depression screening	A structured depression screening should be performed to routinely screen for post-stroke depression.	B-NR^24^
Measuring glycaemia upon ED arrival	First glycaemia measurement should be performed at ED.	B-NR^24^/FeSS^13^
Glycaemia monitoring	Blood glucose levels should be measured at least 12 times during the first 72 h after admission.	B^26^/FeSS^13^
Performing clinical neurological examination and NIHSS documentation	Clinical neurological examination, including documentation of NIHSS score, should be performed in every patient with suspected stroke.	B-NR^24^
Swallowing function screening	Within 24 h after SU admission, swallowing function screening should be performed.	A^26^/FeSS^13^

Definitions of level of evidence applied in this study; level of evidence; (LOE): Level A means high-quality evidence from more than 1 randomized controlled trial (RCT), meta-analyses of high-quality RCTs, or one or more RCTs corroborated by high-quality registry studies; Level B—randomized means moderate-quality evidence from one or more RCTs or meta-analyses of moderate-quality RCTs; Level B—non-randomized means moderate-quality evidence from one or more well-designed, well-executed non-randomized studies, observational studies of registry studies, or meta-analyses of such studies^24^.

FeSS, Fever, Sugar and Swallowing protocol, SU, stroke unit; ED, emergency department.

We collected demographic data, critical steps in the care process, and their timings. We calculated median times for PI: “time to brain imaging” and “time to measuring glycemia upon ED arrival” for all the patients and “door-to-needle (DTN) time” in the subgroup of patients presenting < 4 h after symptom onset (early presenters). In patients admitted > 4 h after symptom onset (late presenters), we assessed median time from ED presentation to antithrombotic administration and to stroke unit (SU) admission. In addition, we determined total length of stay (LOS), defined as time between ED admission and hospital discharge.

### Statistical analysis

Continuous data is presented as mean and SD or median and the 25^th^ (Q1) and 75^th^ (Q3) percentiles. For adherence to QI, mean, SD, and minimum and maximum values are shown at the hospital level. Adherence to QI was assessed using the performance ratio, calculated as the number of patients that received the intervention (numerator) divided by the number of patients for whom the intervention was indicated (denominator). The overall adherence ratios were calculated based on the mean of the individual performance ratios for each hospital or QI. If “no information in the patient record” was indicated, this was analyzed in the same way as “not performed.” We performed a linear regression to identify characteristics of hospitals (numbers of patients with IS treated in 2019 and primary vs. comprehensive stroke centers) as predictors of performance. For time metrics, we determined median and quartiles at the hospital level. SAS version 8.2 was used for analyses.

### Compliance with ethical standards

Ethical approval was obtained from ethical committee of University Hospital Leuven (S64974). The approved protocol was distributed among participating hospitals. Each participating hospital signed a data processing agreement. The study was registered on ClinicalTrials.gov with the identifier: NCT05218135.

## Results

### Patient and hospital characteristics

We included 29 hospitals in this study, representing 46% of the total amount of hospitals in Flanders and Brussels. In 26 of 29 participating hospitals (89.7%), a specialized SU was present. We analyzed data of 870 patients (30 patients/hospital) who met the inclusion and exclusion criteria. Table 2 summarizes demographic and hospital characteristics. Following presentation at the ED, 82.9% of patients were admitted to a specialized SU, but in only 44.8% (*n* = 13) of participating hospitals, this was documented in all the 30 patients.

**Table 2:** Patient and hospital characteristics.

**Patient characteristics (*n* = 870)**	**Mean ±SD or *n* (%)**
Age	73.1 ± 14.0
BMI	26.7 ± 5.0
Unknown	251 (28.9%)
Sex	
Female	418 (48.0%)
Male	452 (52.0%)
NIHSS at admission known	550 (63.2%)
Smoking status	
Smoker	164 (18.9%)
Non-smoker/ex-smoker	512 (58.9%)
Unknown	194 (22.3%)
Diabetes	
Diabetes	203 (23.3%)
Unknown	103 (11.8%)
Hypertension	
Hypertension	520 (59.8%)
Unknown	85 (9.8%)
Hypercholesterolemia	
Hypercholesterolemia	443 (50.9%)
Unknown	119 (13.7%)
Patients with known exact time of symptom onset	598 (68.7%)
Patients treated with thrombolysis	180 (20.7%)
**Hospital characteristics (*****n*** **=** **29)**	
SU available in the hospital	26 (89.7%)
SU beds in 2019	5 ± 4
Number of patients treated for ischemic stroke in 2019 at hospital level	294 ± 207
Comprehensive stroke center	11 (37.9%)

BMI, Body mass Index; SD, standard deviation; n, number; SU, stroke unit.

### Adherence to quality indicator

**Figure 1:**
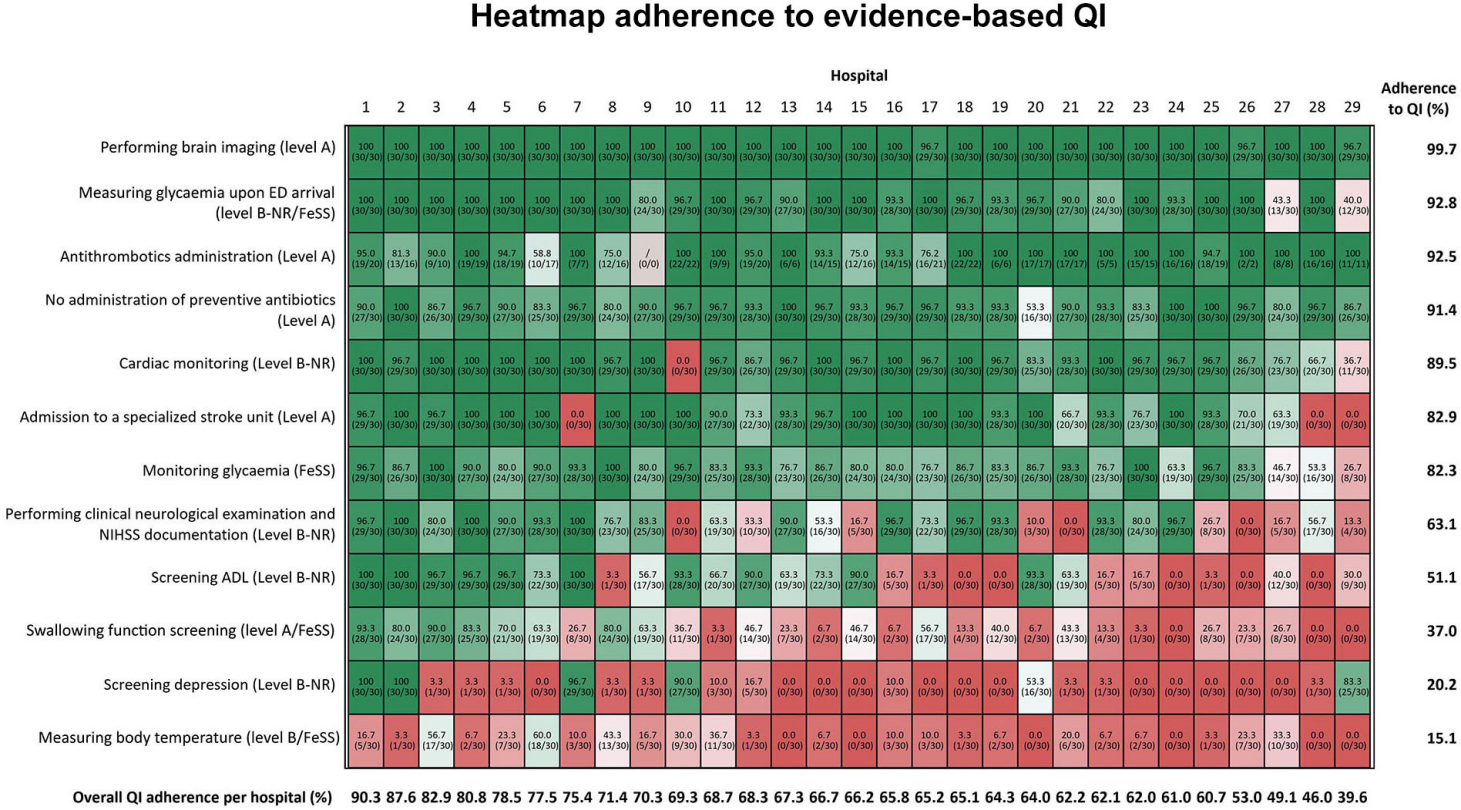
The hospitals' randomization numbers are plotted in columns and QI is depicted in rows of the figure. From top to bottom, the figure is sorted according to total mean adherence ratio for each indicator, which is shown in the right vertical axis. From left to right, the figure is sorted by total mean QI adherence rate for each hospital, which is depicted at the bottom horizontal axis of the figure. The darker red the color of the cells, the poorer the adherence ratio. If no information was found in the record, this was analyzed in a similar way as “not performed”. For all the QI, patient records of all the 30 patients were included in the denominator (*n* = 30), except for antithrombotic administration. For this indicator, only the patients, for whom antithrombotic administration was the primary therapy, were included for the calculation of this QI. FeSS, Fever, Sugar, Swallowing protocol; ADL, activities of daily living; QI, quality indicator.

Adherence to selected QI is shown in Figure 1 and revealed both the inter- and intrahospital variations. The mean overall adherence rate on hospital level was 66.9% and varied from 39.6% to 90.3%. The average indicator adherence rate ranged from 15.1% for measuring body temperature according to the FeSS (Fever, Sugar, and Swallowing) protocol to 99.7% for brain imaging.

We observed better adherence for indicators with higher level of evidence (LOE), visualized at the top of Figure 1: performing brain imaging [99.7 ± 1.0% (96.7–100.0%)], measuring glycemia upon ED arrival [92.8 ± 15.2% (40.0–100.0%)], acute administration of antithrombotic drugs in patients not receiving intravenous thrombolysis [92.5 ± 26.3% (58.8–100%)], no administration of preventive antibiotics [91.4 ± 9.4% (53.3–100%)], and performing cardiac monitoring [89.5 ± 21.7% (0.0–100%)]. Indicators with large interhospital variability were admission to specialized SU [82.9% ± 30.7% (0.0–100%)], monitoring glycemia according to the FeSS protocol [82.3 ± 16.7% (26.7–100.0%)], performing clinical neurological examination and the National Institutes of Health Stroke Scale (NIHSS) documentation [63.1 ± 36.8% (0–100%)], and activities of daily living (ADL) screening [51.1 ± 40.3% (0.0–100.0%)]. Swallowing function screening, screening for depression, and body temperature measurements following FeSS protocol revealed low adherence below 50%, respectively, 37.0 ± 30.4% (0.0–93.3%), 20.2 ± 35.8% (0.0–100%), and 15.1 ± 17.0% (0.0–60%).

In addition, we performed a linear regression to identify a relationship between hospital characteristics and adherence to QI. The number of patients with IS stroke treated in 2019 correlated with performing clinical neurological examinations and the NIHSS documentation (*R*^2^: 0.23; *p* = 0.01), swallowing function screening (*R*^2^: 0.32; *p* = 0.01), and depression screening (*R*^2^: 0.27; *p* = 0.01). In addition, we aimed to identify a potential difference in performance based on type of stroke center. However, we could not determine a difference in the adherence to any of the indicators in primary vs. comprehensive stroke centers.

### Timing of time-sensitive key interventions on the hospital level

**Figure 2:**
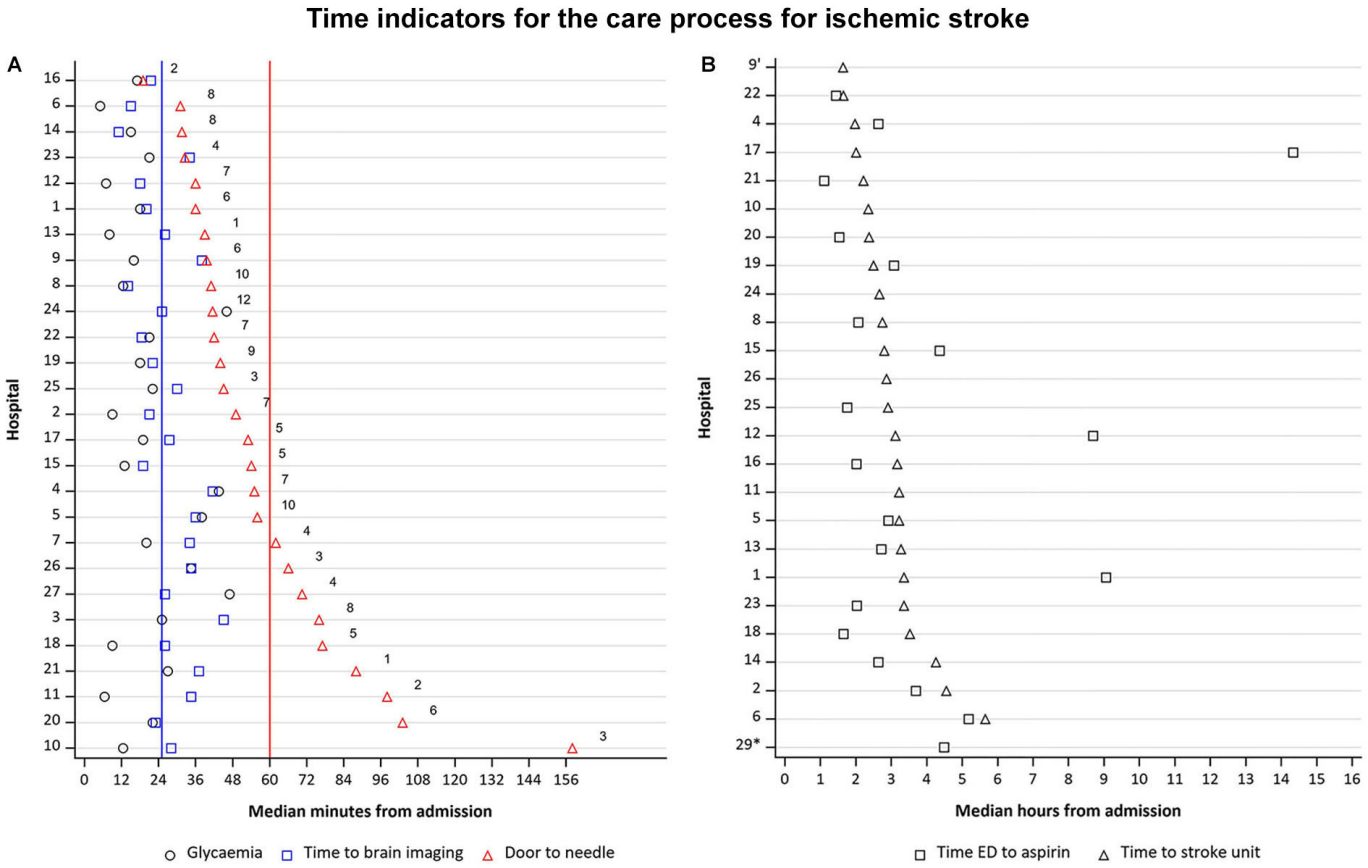
**(A)** Time from ED arrival to glycemia measurement, to imaging, and door-to-needle time, if applicable, for early presenters. 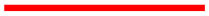 Recommendation for door-to-needle time, according to guidelines. 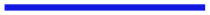 Recommendation for time to brain imaging, according to guidelines Hospitals 28 and 29 are excluded from this figure as they had no data available for door-to-needle time. Numbers in the figure indicate the number of patients who were treated with thrombolysis. *N* = 326. **(B)** Time to SU admission and time to antithrombotic treatment for late presenters. ED, emergency department; SU, stroke unit Hospitals 3,7, 27 and 28 are excluded from this figure as they had no data available for time ED to aspirin and time to SU. ^*^No SU available No data available on time from ED to aspirin administration *N* = 216.

Median time from presentation to brain imaging was 37 min (*n* = 867) and time to glycemia measurement upon arrival in the ED was 24 min (*n* = 807). In 326 (37.5%) early presenters (Figure 2A), with a median onset-to-door time of 84 min (Q1 = 54; Q3 = 138), the median time from presentation to brain imaging (documented in 323 patients, 99.1%) was 26 min (Q1 = 20.5; Q3 = 34.3) with 12 out of 29 hospitals (41.4%) achieving a median of ≤ 25 min, as recommended by guidelines. Time to measurement of glycemia upon ED arrival (documented in 307 patients, 94.2%) was 18.5 min (Q1 = 12.5; Q3 = 23.5). In 153 patients (46.9% of early presenters) who received intravenous thrombolysis, median DTN time was 47 min (Q1 = 39.3; Q3 = 68.3). In 18 hospitals (62.1%), median DTN time was < 60 min and in 13 hospitals (45%), median DTN time was < 45 min.

In 544 late presenters in whom the time from admission to antithrombotic drug administration was known (Figure 2B), median onset-to-door time was 10.0 h (Q1 = 5.9; Q3 = 18.2), median time to initiation of antithrombotic drug was 158 min (Q1 = 2.0; Q3 = 4.4; *n* = 105, 48.6%), and median time to SU admission was 169 min (Q1 = 2.4; Q3 = 3.3; *n* = 182, 84.3%).

**Figure 3:**
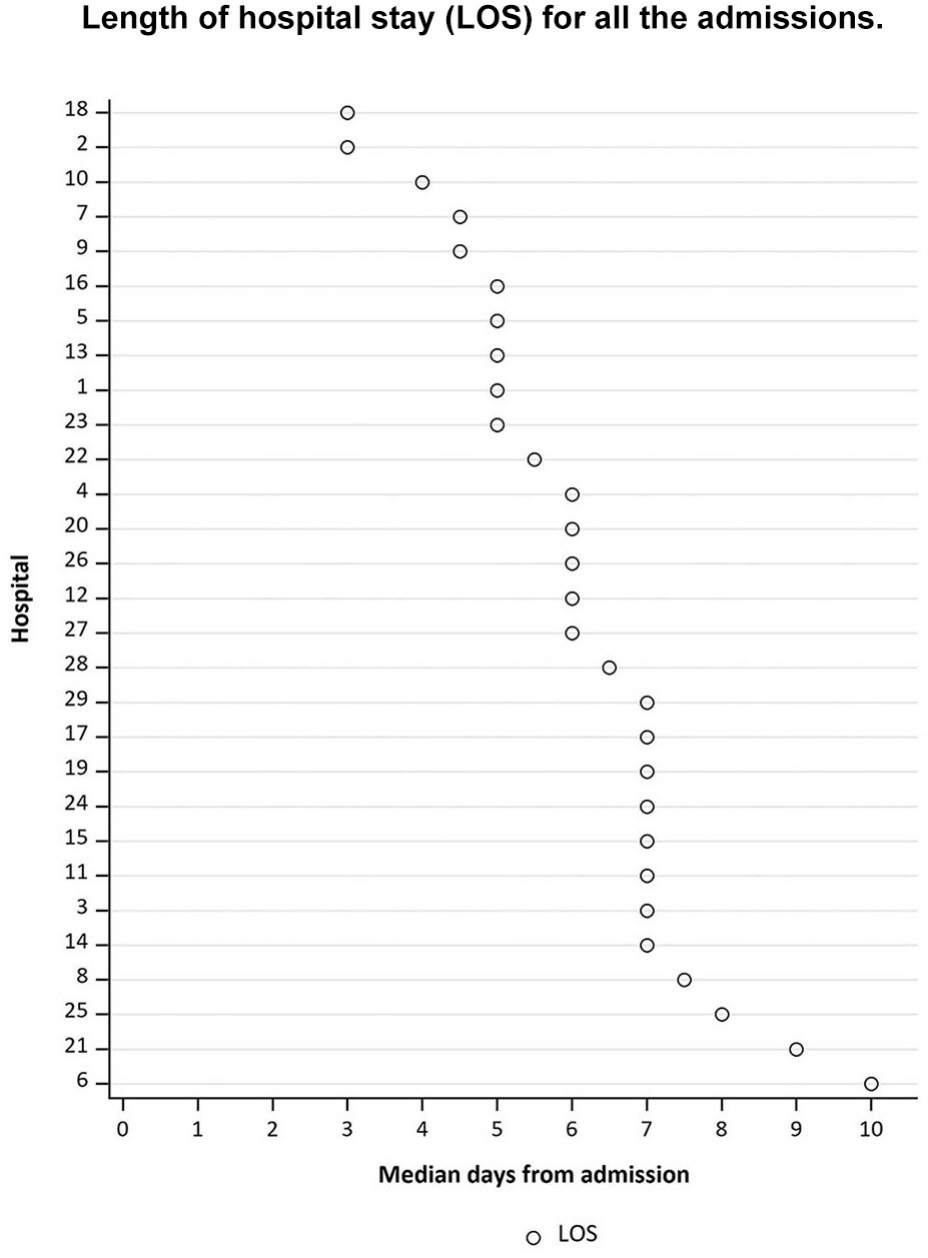
The hospitals' randomization numbers are plotted in the y-axis, sorted from long LOS at the top of the figure, to short LOS at the bottom of the figure. The median days from admission are plotted in the x-axis.

Median LOS for all the patients included in the study was 6 days (Q1 = 5; Q3 = 7), and varied from 3 to 10 days (Figure 3), with 535 (61.5%) patients discharged to home and 151 (17.3%) patients discharged to rehabilitation.

## Discussion

Stroke guidelines aim to guide health professionals on optimal treatment for stroke patients to improve patient outcomes by implementing these recommendations in clinical practice. In this study of 29 hospitals, comprising 870 patients, we found strong adherence and little interhospital variation for QI based on high LOE. Poor adherence with large variations between hospitals was documented for indicators with lower LOE.

Overall mean adherence rate for all the indicators in the present study is higher than previously reported (47% for adherence to stroke-specific guidelines) (29). However, differences in included indicators in these studies may at least partially explain this variation. We found high adherence for following indicators: performing brain imaging, measuring glycemia upon ED arrival, avoiding preventive antibiotics, use of antithrombotic drugs, and performing cardiac monitoring, illustrating robust implementation in the stroke care program. These indicators are based on longstanding guidelines and high LOE, possibly explaining the good adherence in many hospitals (24). Studies reporting on brain imaging determined adherence rates ranging from 85 to 97%, which is in line with the almost 100% in the present study (7, 12, 17). Also, for performing cardiac monitoring and administration of antithrombotic drugs, the results obtained in this study are similar to previous reports (1, 6, 7, 11, 16, 18, 29).

For several other QI, we found large interhospital variability. Most patients were transferred to a specialized SU, but in three hospitals, a SU was not available. There is evidence of a strong treatment effect of SU care on mortality, functional outcome, and quality of life (QoL). Patients treated outside SU are less likely to receive important key interventions such as thrombolysis, multidisciplinary treatment, or prescription of adequate secondary prevention strategies at discharge (11, 30–33). The hospitals without specialized SU also showed low adherence to other quality indicators providing indirect evidence of the importance of admitting patients in SU. Other QI with wide variation was glycemia monitoring and performing clinical neurological examination and the NIHSS documentation. These results clearly indicate that some hospitals have successfully implemented protocols and ensured adherence, while others still need to make progress. For these less well-performing hospitals, the wide variation identified in our study at least suggests that these high adherence rates can be achieved, possibly by quality improvements projects. For ADL screening, a wide adherence range was revealed with 34% of hospitals reaching rates of more than 80%, but 45% of hospitals < 50%. Importance of ADL screening has only been postulated by recent guidelines, which can explain this distinction (24, 34). Potentially, these new guidelines are not yet implemented in protocols and routine clinical practice in some hospitals resulting in poor rates. We selected ADL screening as QI since many stroke survivors suffer from limitations in their ADL, as a result of mental or physical impairment. Proper assessment followed by initiating adequate therapeutic interventions is important since ADL is a predictor for functional status at discharge and duration of rehabilitation (35, 36).

We observed a number of QI with poor adherence, illustrated by an adherence rate of < 50%. These were: swallowing function screening, timely measurement of body temperature according to the FeSS protocol, and depression screening. The adherence rate to swallowing function screening varies in literature between 1 and 92% (1, 11, 16–18, 29, 37–39). This wide range of adherence rates is remarkably since diagnosing and, thereafter, treating of swallowing disorders prevents life-threatening complications as airway obstruction, pneumonia, and malnutrition and is associated with reduced LOS, disability, and mortality (32, 40–42). Therefore, focus on dysphagia and not allowing patients to start eating or drinking before screening is performed have great clinical relevance. Based on the current study, we have no insights in potential reasons for this low adherence, but we aim to focus on potential barriers of implementation in further follow-up. For depression screening, the low rates possibly reflect the existence of only a few guidelines on assessment, treatment, and prevention of post-stroke depression (24, 26, 34). The recommendation to screen for depression was recently added to guidelines following the publication of the first scientific statement from the American Stroke Association on the topic of post-stroke depression in 2017 (24, 43). Translation of these recommendations to daily practice in hospitals included in this study is clearly insufficient. Further improvement is warranted as post-stroke depression that is one of the most common complications after stroke and associated with recurrent vascular events, suboptimal QoL, and mortality (32, 43). The indicator with lowest adherence was timely measurement of body temperature as described in FeSS protocol, with only 2 hospitals accomplishing an adherence rate above 50% (13, 14). Although temperature evaluations were documented in patient records, the number of assessments was less frequent in comparison to the FeSS protocol, which defines strict measurements of temperature every 4 h.

As treating IS is time critical, guidelines state that brain imaging needs to be initiated within 25 min of arrival at the ED (24, 25, 44, 45). For the early presenters, 41% of the hospitals fulfilled this criterion. As the beneficial effect of intravenous thrombolysis declines with time from symptom onset (hence the adage “Time is Brain”), this treatment should be initiated as close as possible to ED admission. We documented median DTN times below 45 min, as suggested in the guidelines, in 45% of the hospitals (34). Improvement projects to identify barriers and to reduce DTN times can assist to increase the amount of hospitals fulfilling these criteria and even further lower these DTN times (46). We identified a relationship between hospital characteristics and some performance measurements as larger hospitals showed better adherence. These findings underscore the importance of organizing stroke care in hospitals with stroke units, which admit a sufficiently large number of patients each year (30, 33, 47–49).

Reports on quality of stroke care are characterized by heterogeneity in the set of QI collected, which hamper comparison of various findings. Since clear guidelines exist, this may sound counterintuitive, but it could also reflect the large number of topics covered by these recommendations, resulting in variations in implementation and monitoring of adherence (24). Monitoring and benchmarking of stroke care could benefit from designing a set of well-defined QI based on guideline recommendations (50, 51). In this study, we identified several stroke care elements with low adherence rates. Therefore, we will develop an improvement plan by providing hospital online learning modules. We prefer to focus on the performance and documentation of the three FeSS interventions (measurement of body temperature and glycemia and swallowing function screening), ADL, and depression screening.

Although this study succeeded in including a large set of patients from a wide variety of hospitals, we acknowledge several limitations. First, we initiated this study in 2020 with the aim to provide an overview of stroke care in Belgium, without interference of a possible modifier as the COVID-19 pandemic. This resulted in the design of a retrospective instead of a prospective study, which is of course preferred. We analyzed patient records of consecutive patients to minimize the effect of this limitation and to avoid selection bias. Second, each of the 29 hospitals collected data for only a limited amount of patients with IS (*n* = 30). We believe that the evaluation of 30 consecutive patient records is sufficient to study the care process at hospital level. Previous studies reporting on stroke care focused on less centers with more patients enabling the visualization of more complete data, but with less information of interhospital variation. Alternatively, data from large stroke registries can be studied to increase the sample size, but typically these reports do not reveal inter- and intrahospital variation, which was the aim of our study. Third, we acknowledge that the study suffers from missing data for several parameters, which may impact our analyses. However, we believe this to represent an important result indicating a lack of full documentation of the variables within the participating hospitals, which can influence the organization of the care processes. We have previously shown that performing patient record analyses repeatedly may improve the completeness of data collection (52). We intend to monitor this in future measurement periods. Last, patients treated with mechanical thrombectomy were excluded during this study, which may have an influence on the included patient population.

## Conclusion

We documented intra- and interhospital variation for various PI and QI in a cohort of 870 patients with IS recruited from 29 hospitals. Hospitals accurately performed recommendations based on some well- and long-established evidence, but showed lower adherence with increased between hospital variation for similarly important and robust quality measurements.

## Data availability statement

The raw data supporting the conclusions of this article will be made available by the authors, without undue reservation.

## Ethics statement

The studies involving human participants were reviewed and approved by Ethical Committee of University Hospital Leuven. Written informed consent for participation was not required for this study in accordance with the national legislation and the institutional requirements.

## Author contributions

CL researched literature and wrote the first draft of the manuscript. RL, KV, EC, and CL conceived the study. CL and EC were involved in protocol development, gaining ethical approval, and hospital recruitment. CL and DS conducted data analysis. All the authors have reviewed and edited the manuscript and approved the final version of the manuscript.

## Conflict of interest

The authors declare that the research was conducted in the absence of any commercial or financial relationships that could be construed as a potential conflict of interest.

## Publisher's note

All claims expressed in this article are solely those of the authors and do not necessarily represent those of their affiliated organizations, or those of the publisher, the editors and the reviewers. Any product that may be evaluated in this article, or claim that may be made by its manufacturer, is not guaranteed or endorsed by the publisher.

## References

[1] Hsieh FI Jeng JS Chern CM Lee TH Tang SC Tsai LK. Quality improvement in acute ischemic stroke care in Taiwan: the breakthrough collaborative in stroke. PLoS ONE. (2016) 11:e0160426. 10.1371/journal.pone.0160426 27487190 PMC4972387

[2] Kieny M-PE Timothy G Scarpetta S Kelley ET Klazinga N Forde I. Delivering Quality Health Services: A Global Imperative for Universal Health Coverage. Geneva: World Health Organization; Organisation for Economic Co-operation Development, the World Bank (2018). Licence: CC BY-NC-SA 3.0 IGO. Available online at: https://apps.who.int/iris/bitstream/handle/10665/272465/9789241513906-eng.pdf?sequence=1&isAllowed=y

[3] Atsma F Elwyn G Westert G. Understanding unwarranted variation in clinical practice: a focus on network effects, reflective medicine and learning health systems. Int J Qual Health Care. (2020) 32:271–4. 10.1093/intqhc/mzaa023 32319525 PMC7270826

[4] Asch SM Kerr EA Keesey J Adams JL Setodji CM Malik S. Who is at greatest risk for receiving poor-quality health care? N Engl J Med. (2006) 354:1147–56. 10.1056/NEJMsa044464 16540615

[5] Andrew NE Middleton S Grimley R Anderson CS Donnan GA Lannin NA. Hospital organizational context and delivery of evidence-based stroke care: a cross-sectional study. Implement Sci. (2019) 14:6. 10.1186/s13012-018-0849-z 30658654 PMC6339367

[6] Mohammed M Zainal H Tangiisuran B Harun SN Ghadzi SM Looi I. Impact of adherence to key performance indicators on mortality among patients managed for ischemic stroke. Pharm Pract (Granada). (2020) 18:1760. 10.18549/PharmPract.2020.1.1760 32256900 PMC7092711

[7] Ayis SA Coker B Bhalla A Wellwood I Rudd AG Di Carlo A. Variations in acute stroke care and the impact of organised care on survival from a European perspective: the European Registers of Stroke (EROS) investigators. J Neurol Neurosurg Psychiatry. (2013) 84:604–12. 10.1136/jnnp-2012-303525 23385847

[8] Muñoz Venturelli P Li X Middleton S Watkins C Lavados PM Olavarría VV. Impact of evidence-based stroke care on patient outcomes: a multilevel analysis of an international study. J Am Heart Assoc. (2019) 8:e012640. 10.1161/JAHA.119.012640 31237173 PMC6662356

[9] Donnellan C Sweetman S Shelley E. Health professionals' adherence to stroke clinical guidelines: a review of the literature. Health Policy. (2013) 111:245–63. 10.1016/j.healthpol.2013.05.002 23727250

[10] Muñoz Venturelli P Robinson T Lavados PM Olavarría VV Arima H Billot L. Regional variation in acute stroke care organisation. J Neurol Sci. (2016) 371:126–30. 10.1016/j.jns.2016.10.026 27871433

[11] Haas K Rücker V Hermanek P Misselwitz B Berger K Seidel G. Association between adherence to quality indicators and 7-day in-hospital mortality after acute ischemic stroke. Stroke. (2020) 51:3664–72. 10.1161/STROKEAHA.120.029968 33040703

[12] Bray BD Ayis S Campbell J Hoffman A Roughton M Tyrrell PJ. Associations between the organisation of stroke services, process of care, and mortality in England: prospective cohort study. BMJ. (2013) 346:f2827. 10.1136/bmj.f2827 23667071 PMC3650920

[13] Middleton S McElduff P Ward J Grimshaw JM Dale S D'Este C. Implementation of evidence-based treatment protocols to manage fever, hyperglycaemia, and swallowing dysfunction in acute stroke (QASC): a cluster randomised controlled trial. Lancet. (2011) 378:1699–706. 10.1016/S0140-6736(11)61485-2 21996470

[14] Dello S Lemmens R Demeestere J Michiels D Wellens L Weltens C. A nurse-led multicomponent intervention supported by advanced electronic health records to improve the acute management of stroke patients: a pre-and post-intervention study. Int J Nurs Stud Advances. (2021) 3:100023. 10.1016/j.ijnsa.2021.100023 PMC11080281 38746710

[15] Middleton S Pfeilschifter W. International translation of fever, sugar, swallow protocols: the quality in acute stroke care Europe project. Int J Stroke. (2020) 15:591–4. 10.1177/1747493020915130 32299312

[16] Li X Wang C Rehman S Wang X Zhang W Su S. Setting performance benchmarks for stroke care delivery: which quality indicators should be prioritized in quality improvement; an analysis in 500,331 stroke admissions. Int J Stroke. (2021) 16:727–37. 10.1177/1747493020958608 32957865

[17] Hall RE Khan F Bayley MT Asllani E Lindsay P Hill MD. Benchmarks for acute stroke care delivery. Int J Qual Health Care. (2013) 25:710–8. 10.1093/intqhc/mzt069 24141011 PMC3842126

[18] Wang Y Li Z Zhao X Liu L Wang C Wang C. Evidence-based performance measures and outcomes in patients with acute ischemic stroke. Circ Cardiovasc Qual Outcomes. (2018) 11:e001968. 10.1161/CIRCOUTCOMES.115.001968 30557048

[19] van de Vijsel AR Heijink R Schipper M. Has variation in length of stay in acute hospitals decreased? Analysing trends in the variation in LOS between and within Dutch hospitals. BMC Health Serv Res. (2015) 15:438. 10.1186/s12913-015-1087-6 26423895 PMC4590267

[20] Viglianti EM Bagshaw SM Bellomo R McPeake J Wang XQ Seelye S. Hospital-level variation in the development of persistent critical illness. Intensive Care Med. (2020) 46:1567–75. 10.1007/s00134-020-06129-9 32500182 PMC7444658

[21] Kringos DS Sunol R Wagner C Mannion R Michel P Klazinga NS. The influence of context on the effectiveness of hospital quality improvement strategies: a review of systematic reviews. BMC Health Serv Res. (2015) 15:277. 10.1186/s12913-015-0906-0 26199147 PMC4508989

[22] Coles E Anderson J Maxwell M Harris FM Gray NM Milner G. The influence of contextual factors on healthcare quality improvement initiatives: a realist review. Syst Rev. (2020) 9:94. 10.1186/s13643-020-01344-3 32336290 PMC7184709

[23] Baatiema L Otim ME Mnatzaganian G. de-Graft Aikins A, Coombes J, Somerset S. Health professionals' views on the barriers and enablers to evidence-based practice for acute stroke care: a systematic review. Implement Sci. (2017) 12:74. 10.1186/s13012-017-0599-3 28583164 PMC5460544

[24] Powers WJ Rabinstein AA Ackerson T Adeoye OM Bambakidis NC Becker K. Guidelines for the early management of patients with acute ischemic stroke: 2019 update to the 2018 guidelines for the early management of acute ischemic stroke: a guideline for healthcare professionals from the American Heart Association/American Stroke Association. Stroke. (2019) 50:e344–418. 10.1161/STR.0000000000000211 31662037

[25] Powers WJ. Acute ischemic stroke. N Engl J Med. (2020) 383:252–60. 10.1056/NEJMcp1917030 32668115

[26] Boulanger JM Lindsay MP Gubitz G Smith EE Stotts G Foley N. Canadian stroke best practice recommendations for acute stroke management: prehospital, emergency department, and acute inpatient stroke care, 6th edition, update 2018. Int J Stroke. (2018) 13:949–84. 10.1177/1747493018786616 30021503

[27] Characteristics of clinical indicators. QRB Qual Rev Bull. (1989) 15:330–9. 10.1016/S0097-5990(16)30313-X 2512521

[28] Becker M Breuing J Nothacker M Deckert S Brombach M Schmitt J. Guideline-based quality indicators-a systematic comparison of German and international clinical practice guidelines. Implement Sci. (2019) 14:71. 10.1186/s13012-019-0918-y 31288828 PMC6617919

[29] Abanto C Ulrich AK Valencia A Dueñas V Montano S Tirschwell D. Adherence to American Heart Association/American Stroke Association Clinical Performance Measures in a Peruvian Neurological Reference Institute. J Stroke Cerebrovasc Dis. (2020) 29:105285. 10.1016/j.jstrokecerebrovasdis.2020.105285 33066929 PMC7575824

[30] Cadilhac DA Kilkenny MF Andrew NE Ritchie E Hill K Lalor E. Hospitals admitting at least 100 patients with stroke a year should have a stroke unit: a case study from Australia. BMC Health Serv Res. (2017) 17:212. 10.1186/s12913-017-2150-2 28302181 PMC5356228

[31] Cadilhac DA Andrew NE Lannin NA Middleton S Levi CR Dewey HM. Quality of acute care and long-term quality of life and survival: the Australian Stroke Clinical Registry. Stroke. (2017) 48:1026–32. 10.1161/STROKEAHA.116.015714 28258253

[32] Green TL McNair ND Hinkle JL Middleton S Miller ET Perrin S. Care of the patient with acute ischemic stroke (Posthyperacute and Prehospital Discharge): update to 2009 comprehensive nursing care scientific statement: a scientific statement from the American Heart Association. Stroke. (2021) 52:e179–e97. 10.1161/STR.0000000000000357 33691469

[33] Ringelstein EB Chamorro A Kaste M Langhorne P Leys D Lyrer P. European Stroke Organisation recommendations to establish a stroke unit and stroke center. Stroke. (2013) 44:828–40. 10.1161/STROKEAHA.112.670430 23362084

[34] Powers WJ Rabinstein AA Ackerson T Adeoye OM Bambakidis NC Becker K. 2018 Guidelines for the early management of patients with acute ischemic stroke: a guideline for healthcare professionals from the American Heart Association/American Stroke Association. Stroke. (2018) 49:e46–e110. 10.1161/STR.0000000000000158 29367334

[35] Collin C Wade DT Davies S Horne V. The barthel ADL index: a reliability study. Int Disabil Stud. (1988) 10:61–3. 10.3109/09638288809164103 3403500

[36] Legg LA Lewis SR Schofield-Robinson OJ Drummond A Langhorne P. Occupational therapy for adults with problems in activities of daily living after stroke. Cochrane Database Syst Rev. (2017) 7:Cd003585. 10.1002/14651858.CD003585.pub3 28721691 PMC6483548

[37] Hoffmeister L Lavados PM Comas M Vidal C Cabello R Castells X. Performance measures for in-hospital care of acute ischemic stroke in public hospitals in Chile. BMC Neurol. (2013) 13:23. 10.1186/1471-2377-13-23 23496941 PMC3599613

[38] Liu ZY Zhang XP Mo MM Ye RC Hu CX Jiang MQ. Impact of the systematic use of the volume-viscosity swallow test in patients with acute ischaemic stroke: a retrospective study. BMC Neurol. (2020) 20:154. 10.1186/s12883-020-01733-0 32334559 PMC7183112

[39] Joundi RA Martino R Saposnik G Giannakeas V Fang J Kapral MK. Predictors and outcomes of dysphagia screening after acute ischemic stroke. Stroke. (2017) 48:900–6. 10.1161/STROKEAHA.116.015332 28275200

[40] Perry L Love CP. Screening for dysphagia and aspiration in acute stroke: a systematic review. Dysphagia. (2001) 16:7–18. 10.1007/PL00021290 11213249

[41] Carnaby G Hankey GJ Pizzi J. Behavioural intervention for dysphagia in acute stroke: a randomised controlled trial. Lancet Neurol. (2006) 5:31–7. 10.1016/S1474-4422(05)70252-0 16361020

[42] Xi X Li H Wang L Yin X Zeng J Song Y. How demographic and clinical characteristics contribute to the recovery of post-stroke dysphagia? Medicine (Baltimore). (2021) 100:e24477. 10.1097/MD.0000000000024477 33530262 PMC7850691

[43] Towfighi A Ovbiagele B El Husseini N Hackett ML Jorge RE Kissela BM. Poststroke depression: a scientific statement for healthcare professionals from the American Heart Association/American Stroke Association. Stroke. (2017) 48:e30–43. 10.1161/STR.0000000000000113 27932603

[44] Bonadio W Beck C Mueller A. Impact of CT scanner location on door to imaging time for emergency department stroke evaluation. Am J Emerg Med. (2020) 38:309–10. 10.1016/j.ajem.2019.158398 31488337

[45] Zaidi SF Shawver J Espinosa Morales A Salahuddin H Tietjen G Lindstrom D. Stroke care: initial data from a county-based bypass protocol for patients with acute stroke. J Neurointerv Surg. (2017) 9:631–5. 10.1136/neurintsurg-2016-012476 27342763 PMC5520240

[46] Mikulik R Bar M Cernik D Herzig R Jura R Jurak L. Stroke 20 20: implementation goals for intravenous thrombolysis. Eur Stroke J. (2021) 6:151–9. 10.1177/23969873211007684 34414290 PMC8370063

[47] Lee KJ Kim JY Kang J Kim BJ Kim SE Oh H. Hospital volume and mortality in acute ischemic stroke patients: effect of adjustment for stroke severity. J Stroke Cerebrovasc Dis. (2020) 29:104753. 10.1016/j.jstrokecerebrovasdis.2020.104753 32151475

[48] Hall RE Fang J Hodwitz K Saposnik G Bayley MT. Does the Volume of Ischemic Stroke Admissions Relate to Clinical Outcomes in the Ontario Stroke System? Circ Cardiovasc Qual Outcomes. (2015) 8(6 Suppl. 3):S141–7. 10.1161/CIRCOUTCOMES.115.002079 26515202

[49] Saposnik G Baibergenova A O'Donnell M Hill MD Kapral MK Hachinski V. Hospital volume and stroke outcome: does it matter? Neurology. (2007) 69:1142–51. 10.1212/01.wnl.0000268485.93349.58 17634420

[50] Norrving B Barrick J Davalos A Dichgans M Cordonnier C Guekht A. Action plan for stroke in Europe 2018-2030. Eur Stroke J. (2018) 3:309–36. 10.1177/2396987318808719 31236480 PMC6571507

[51] Organisation ES. Key performance indicators: to monitor and facilitate change of the SAP-E implementation. Available online at: https://actionplan.eso-stroke.org/key-performance-indicators/. (accessed on June30, 2022).

[52] Coeckelberghs E Vanhaecht K Seys D Cox B Bislenghi G Wolthuis AM. A breakthrough improvement collaborative significantly reduces hospital stay after elective colectomy for cancer across a healthcare system. Ann Surg. (2022). 10.1097/SLA.0000000000005646 35916138 PMC9534055

