# Supplementary material for: Variation in stroke care at the hospital level: A cross-sectional multicenter study
Source: Front Neurol. 2022 Oct 13;13:1004901. doi: 10.3389/fneur.2022.1004901 (PMC9606690; doi:10.3389/fneur.2022.1004901)
Supplement: Supplementary file 1 [file Data_Sheet_1.PDF]

Supplementary Material

1 Supplementary Data

N/A

2 Supplementary Figures and Tables

2.1 To quantify the variability between hospitals, we provide a table to visualize the Interquartile range (IQR) of all parameters from low to high:

**Quality Indicator**	Q1	Q3	IQR
First brain imaging	100	100	0
Antithrombotics administration	93.3	100	6.7
Cardiac monitoring	93.3	100	6.7
Measuring glycaemia upon ED arrival	93.3	100	6.7
No administration of preventive antibiotics	90	96.7	6.7
Screening depression	0	10	10
Monitoring glycaemia	80	93.3	13.3
Measuring temperature	3.3	23.3	20
Admission to a specialized stroke unit	76.7	100	23.3
Swallowing function screening	6.7	63.3	56.7
Performing clinical neurological examination and NIHSS documentation	26.7	93.3	66.7
Screening ADL	3.3	93.3	90

Q1 = 25th percentile, Q3 = the 75th percentile, IQR = interquartile range; Calculations were based on the mean of the individual performance ratios for each hospital.

2.2

A linear regression was used to identify characteristics of hospitals (numbers of IS patients treated in 2019 and primary vs comprehensive stroke centres) as predictors of performance.

Quality indicator	R^2	P-value
First brain imaging	0.001	0.9
No administration of preventive antibiotics	0.03	0.4
Measuring glycaemia upon ED arrival	0.05	0.5
Admission to a specialize stroke unit	0.05	0.9
Antithrombotics administration	0.06	0.4
Monitoring glycaemia	0.07	0.2
Cardiac monitoring	0.08	0.2
Measuring temperature	0.09	0.3
Screening ADL	0.14	0.1
Screening depression	0.23	0.01*
Performing clinical and neurological examination and NIHSS documentation	0.23	0.04*
Swallowing function screening	0.30	0.01*
